# How Did Seal Lice Turn into the Only Truly Marine Insects?

**DOI:** 10.3390/insects13010046

**Published:** 2021-12-31

**Authors:** María Soledad Leonardi, José E. Crespo, Florencia Soto, Claudio R. Lazzari

**Affiliations:** 1 IBIOMAR–CONICET, Puerto Madryn 9120, Argentina; leonardi@cenpat-conicet.gob.ar (M.S.L.); fsoto@cenpat-conicet.gob.ar (F.S.); 2 Instituto de Ecología, Genética y Evolución (IEGEBA), CONICET–Universidad de Buenos Aires, Buenos Aires 1428, Argentina; crespo@ege.fcen.uba.ar; 3 Institut de Recherche sur la Biologie de l’Insecte, UMR CNRS 7261–University of Tours, 37200 Tours, France

**Keywords:** adaptation, Anoplura, Echinophthiriidae, extreme environments

## Abstract

**Simple Summary:**

Sucking lice are permanent and obligate ectoparasites throughout their whole life cycle. Echinophthiriids escorted their mammal hosts during their passage from fully terrestrial to amphibian life. Seal lice synchronize their reproduction cycle with that of their mammalian hosts. Echinophthiriids tolerate long immersion periods and extreme hydrostatic pressures. Diving lice can reach kilometers under the surface and survive, during the months their hosts remain in the open ocean. In the present work, we describe and discuss how some of these adaptations allow seal lice to cope with the amphibious habits of their hosts and how they can help us to understand why insects are so rare in the ocean.

**Abstract:**

Insects are the most evolutionarily and ecologically successful group of living animals, being present in almost all possible mainland habitats; however, they are virtually absent in the ocean, which constitutes more than 99% of the Earth’s biosphere. Only a few insect species can be found in the sea but they remain at the surface, in salt marshes, estuaries, or shallow waters. Remarkably, a group of 13 species manages to endure long immersion periods in the open sea, as well as deep dives, i.e., seal lice. Sucking lice (Phthiraptera: Anoplura) are ectoparasites of mammals, living while attached to the hosts’ skin, into their fur, or among their hairs. Among them, the family Echinophthiriidae is peculiar because it infests amphibious hosts, such as pinnipeds and otters, who make deep dives and spend from weeks to months in the open sea. During the evolutionary transition of pinnipeds from land to the ocean, echinophthiriid lice had to manage the gradual change to an amphibian lifestyle along with their hosts, some of which may spend more than 80% of the time submerged and performing extreme dives, some beyond 2000 m under the surface. These obligate and permanent ectoparasites have adapted to cope with hypoxia, high salinity, low temperature, and, in particular, conditions of huge hydrostatic pressures. We will discuss some of these adaptations allowing seal lice to cope with their hosts’ amphibious habits and how they can help us understand why insects are so rare in the ocean.

## 1. Introduction

When one searches for information about marine insects, the literature usually refers to sea-skaters, a small group of Heteroptera of the family Gerridae (“water-striders”), belonging to the genus *Halobates* [1]. Scientists have identified 46 different species, which live in association with the bi-dimensional world of the sea surface [2]. Only five species of *Halobates* live in the open ocean. It is worth mentioning, however, that sea-skaters remain on the surface and never dive below it. As a consequence, they are not truly exposed to marine conditions, even though the sea surface can also be hostile because of the exposure to UV rays, and passive displacement. Technically speaking, *Halobates* should be considered terrestrial insects that live in the open ocean, not so different from their relatives skating on the surface of ponds on the mainland.

Insects first appeared more than 420 Mya during the Silurian–Ordovician epoch and, during the next 300 million years, they dispersed and diversified, colonizing nearly every available mainland habitat. Intriguingly, the most ecologically and evolutionarily successful group of organisms on Earth is virtually absent from the greatest available habitat, i.e., the ocean, which constitutes more than 99% of our biosphere. This lack of insects in the ocean, as well as their occasional occurrence in marine ecosystems, contrasts with their richness on land, leading to a variety of scientific hypotheses and assumptions, which we shall explore further in this paper [3]. Yet, there exists a particular group of insects that managed to survive underwater at great depths during long immersion periods, i.e., seal lice.

Lice (of the order Phthiraptera) are the only group of insects that have become obligate and permanent parasites throughout their entire life cycle, living as ectoparasites among the feathers, fur, or hairs of vertebrate hosts [4,5]. Throughout their evolutionary history, sucking lice (suborder Anoplura) have established associations and co-evolved with mammals, being present in most Mammalian genera, with the exception of those belonging to the orders Monotremata, Cetacea, Sirenia, Pholidota, Edentata, and Proboscidea. Across the great diversity of anopluran lice, the family Echinophthiriidae shows the unique characteristic of infesting amphibious hosts, such as pinnipeds (walruses, seals, and sea lions) and the North American river otter [6,7].

Pinnipeds are diving mammals and many of them forage at significant depths [8]. The most extraordinary diver is the southern elephant seal, which can dive more than 2000 m deep [9]. On the other hand, during the feeding periods (i.e., most of the year), pinnipeds can spend several months in the open sea [10] without returning ashore. Despite the extreme constraints imposed by these habits on echinophthiriid lice, they have managed to adapt to the amphibian biology of their hosts [11]. The survival of an originally terrestrial louse in the deeps of the ocean implies that this insect gradually evolved to tolerate the particular physical conditions of extreme environments, such as high hydrostatic pressure, hypoxia, low temperature, and high salinity.

The underlying mechanisms that allow echinophthiriids to live in association with deep-diving hosts have only recently started to be investigated. This review provides a critical discussion of the state of knowledge about the adaptations of echinophthiriids to survive where no other known insect is capable of surviving. This synthesis is relevant and timely because, until recently, there was a widespread belief that the ectoparasitic lice that live on semi-aquatic mammals would perish if their hosts went to sea. The discovery of adult elephant seals ashore in Antarctica with living adult lice clinging to their bodies has finally disproved this theory [12], meaning that these insects have traveled with their host, probably for months, in the open sea and survived. This finding made us think about echinophthiriids’ unique adaptations to withstand the harsh environments of the ocean. It also pushes us to forsake the notion that insects are not naturally suited to surviving in the ocean.

## 2. Materials and Methods

### 2.1. Evolution

According to molecular and paleontological data, pinnipeds diverged from their carnivorous ancestors about 45 Mya, with the separation of the Feliformia and the Caniformia [13]. Molecular analysis also supports the monophyly of the Pinnipedia, with a basal split between Otariidea (sea lions, fur seals, and walruses) and Phocidae (seals) [14]. Evidence suggests a North American origin for pinnipeds, which was followed by a Pacific dispersal of otariids into the Southern hemisphere and an Atlantic dispersal for phocids. During the colonization of the marine environment, pinnipeds lost most of their parasites [15]. Yet, the fact that pinnipeds kept their contact with the terrestrial environment, allowed some parasites like echinophthiriid lice to accompany this evolutionary process [15,16]. The family Echinophthiriidae comprises five genera and 13 species (Table 1), including *Antarctophthirus,* the ectoparasites of sea lions, Antarctic seals, the northern fur seal, and the walrus; *Echinophthirius* from true seals in the Northern hemisphere; *Latagophthirus* from the North American river otter; *Lepidophthirus* from elephant and monk seals; and *Proechinophthirus* from northern and southern fur seals [6,7,17].

**Figure 1:**
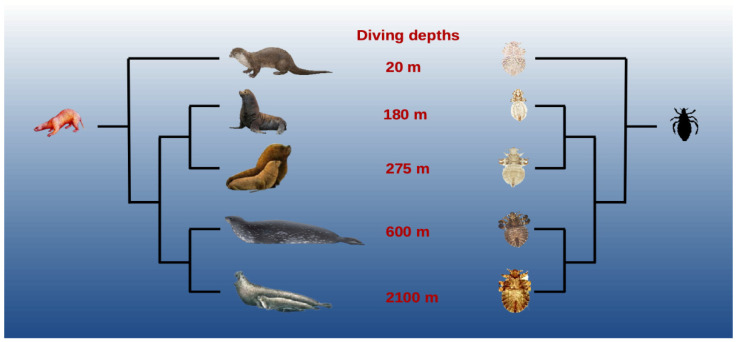
Schematic phylogenetic tree comparing the evolutionary histories of pinnipeds (**left**) and their lice (**right**), modified from Leonardi et al. (2019). Host-louse associations: 1—North American river otter—*Latagophthirus rauschi*; 2—Northern fur seal—*Proechinophthirus fluctus*; 3—Southern sea lion—*Antarctophthirus microchir*; 4—Weddell seal—*A. carlinii*; 5—Southern elephant seal—*Lepidophthirus macrorhini*. Seal images are from Pieter Folkens and the NOAA; *Le. macrorhini* and *La. rauschi* photos are from phthiraptera.org.

A phylogenomic analysis, including *A. microchir* from Southern and Australian sea lions, *A. carlinii* from Weddell seals, *A. lobodontis* from crabeater seals, *A. ogmorhini* from leopard seals, *L. macrorhini* from southern elephant seals, and *P. fluctus* from the northern fur seal, supports the monophyletic origin of the echinophthiriids and the terrestrial origin of this host–parasite association (Figure 1) [11]. These results agree with the pioneering ideas of Kim [4,18]. Based on morphological phylogenetic analysis, Kim was the first to suggest that the terrestrial ancestors of pinnipeds were already infested by ancestral sucking lice. Therefore, lice adapted to the new environmental conditions imposed by their hosts. This is likely one of the primary reasons why lice became the only insects to colonize the deep sea, probably acquiring unique morphological, physiological, behavioral, and ecological adaptations in the process to cope with the amphibious lifestyle of their hosts. Next, we discuss the main adaptations that allowed lice to coevolve alongside their hosts.

### 2.2. Morphological Adaptations

Echinophthiriids present some unique morphological adaptations for underwater life. Firstly, all species have the tibia-tarsi of second and third pairs of legs that are strongly adapted to clinging. The first pair of legs in most species is smaller and more slender than the others. Probably, these legs play a sensory role in insects where, according to the literature, eyes are absent [4,19,20]. However, the first pair of legs of *L. macrorhini* is robust, and the tarsal claws are modified into well-developed hooks [21]. It has been suggested that this species utilizes its claws to perforate the skin and dig into the host epidermis, in order to stay attached during elephant seal molting [21]. Regarding the absence of eyes, a series of studies in different species is required to determine the presence of specific structures or pigments capable of detecting light.

Secondly, according to Kim (see Figure 343 in [22]), the louse spiracles present an elaborated closing device that could have a double function, i.e., to preserve the atmospheric air into the tracheal system and to prevent the entry of seawater during immersions. However, due to the extremely high hydrostatic pressure seen during deep dives, the tracheal system may entirely collapse [23]; some oxygen could be conserved at a cellular level, either dissolved or associated with (as yet unknown) respiratory pigments. Thus, the elaborated system for closing spiracles would be more related to avoiding the entry of water, rather than retaining air in the tracheal system.

Finally, the abdomens of seal lice are membranous and considerably thicker than the typical Anopluran abdomen [19]. It has been identified for *A. carlinii* that the ventral surface cuticle is at least half as thin as the dorsal side and it is especially thin in the head. [19]. A thin cuticle could enable gas exchange and cutaneous respiration, a possibility that remains to be investigated. We will discuss this point in more detail later on.

**Figure 2:**
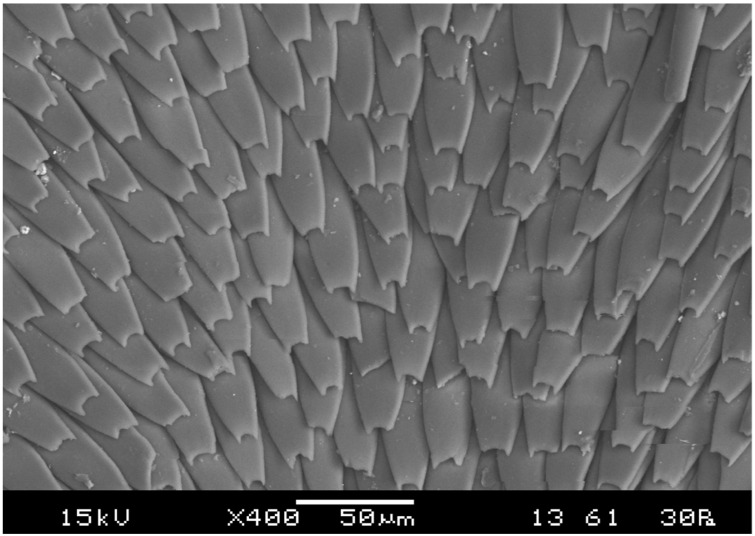
Scanning electron micrography of a female *Lepidophthirus macrorhini,* depicting the disposition of scales.

Scales, or specialized and modified spines [24], are a distinctive feature of echinophthiriids (Figure 2), and their density and size increase as they develop [19,20,25,26]. The initial nymphal stage, which remains in the case of non-swimming juvenile hosts, is devoid of scales [20,22]. The specific role of scales has been discussed many years ago, and two different possible functions have been proposed, both related to adaptations for surviving underwater [22,27,28]. Murray [26] postulated that scales would protect the cuticle from mechanical damage (e.g., by high hydrostatic pressure) and against desiccation, whereas Hinton [29] proposed that scales could form a “plastron” (i.e., a physical gill formed by a thin layer of air, retained by hydrophobic structures) making underwater respiration possible. These two hypotheses are not mutually exclusive, and recent studies did provide support to both of them. On the one hand, Leonardi and Lazzari [30] reported that sea lion lice showed higher survival rates and shorter recovery times when they were submerged for variable time periods, between 1 to 15 days, in normoxic rather than in hypoxic water, supporting aquatic respiration and, by extension, Hinton’s hypothesis. On the other hand, experiments aimed at determining tolerance to high hydrostatic pressure also revealed that adults, which have their bodies fully covered with scales, performed better than nymphs with fewer scales over their bodies [23]. It should be noted, however, that the effects of high hydrostatic pressure would include collapsing air-filled cavities, as tracheal tubes, and, when the pressure is very high, affecting cellular and molecular integrity. Since the lice body is mostly incompressible, it is hard to make a link between scales and tolerance to high pressure, as suggested by Murray [26].

### 2.3. Reproductive Synchronization with Hosts

One of the greatest constraints for echinophthiriids is that their eggs do not survive underwater [30,31]. Consequently, lice reproduction can only occur during those periods when hosts remain on land for enough time, i.e., during their reproduction and molting season. So, the reproductive events and the number of lice generations per year are constrained by the haul-out behavior of their hosts. Indeed, there is an adaptive reproductive schedule of seal lice according to the biology and ecology of their hosts [28]. For instance, in the case of *A. microchir* from South American sea lions, the reproductive season is the only moment of the life cycle when the host spends enough time ashore, and only newborn pups remain outside the water long enough to allow lice to reach the imaginal state [27,30]. Instead, in the case of *A. lobodontis* from the crabeater seal, reproduction and transmission would only be possible with juvenile hosts [32].

### 2.4. Tolerance to Immersion

**Figure 3:**
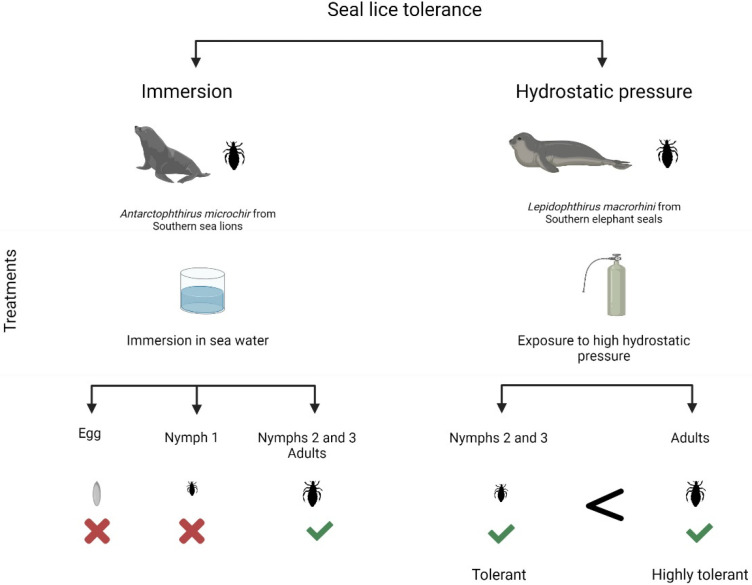
Experimental design and main results testing the tolerance to immersion (**left**) and high hydrostatic pressure (**right**). **✓** indicates survival; **✕** death.

A series of experiments have been conducted on nymphs and adults, to evaluate lice survival under different conditions of immersion and temperature, using the protocol depicted in Figure 3 (for details, see [30]). It was observed that the first nymphs (N1) were unable to survive underwater but the rest of the instars and adults tolerated submersions lasting for several days [30]. Previous contributions by Murray and Nicholls [33] had already reported the death of eggs and the survival in seawater of advanced nymphs and adults; however, N1 were not included in their experiment. According to the findings of a recent study, N1 can only withstand immersion for a few days. The reduced tolerance to immersions of N1 compared to more advanced instars explains the reduction of N1 in the South American sea-lion pup population when they start to swim, as alleged by Leonardi and Lazzari [30]. Murray and co-workers had previously arrived at a similar conclusion [31,33], as well as Kim [22], from the absence of N1 in old pups and adult pinnipeds on northern fur seals. The incapacity of N1 to survive underwater was suggested to be associated with the absence of abdominal scales [20,22,27], which are abundantly present in the tolerant instars.

When seal lice emerge in seawater, they experience a reduction in oxygen availability, and a rapid and large drop in temperature (20–25 °C difference between air and water in summer), as well as an increase in hydrostatic pressure. It has been reported that contact with seawater triggers reflex immobility (akinesis) in *A. microchir* and in *L. macrorhini*, which is immediate (in seconds) in the former and requires several minutes in the latter [23,30]. Thus, it seems that the tolerance to immersion depends on a reflex reduction in metabolism and activity (i.e., quiescence) triggered by the physical contact of lice with seawater. This rapid response would help to spare energy, nutrients, and oxygen, consequently allowing the survival of lice for a long time underwater. Furthermore, their differential survival when submerged in normoxic or in hypoxic water [30] strongly suggests that echinophthiriids would be able to exchange gases with the surrounding water, a capacity never before reported in the group. The adaptations and mechanisms that underpin this ability are currently unknown, and more anatomical and physiological research is required.

### 2.5. Tolerance to Hydrostatic Pressure

In another series of experiments, depicted in Figure 3, it was found that lice from elephant seals can tolerate hydrostatic pressures equivalent to 2000 m in depth [23], which represents a depth equivalent to seven times the Eiffel Tower or the Empire State Building beneath the surface of the sea. Serendipitously, a louse was observed to survive to a pressure of 450 kg cm^−2^ (eq. 4.5 km in depth) when accidentally exposed to this hydrostatic pressure for some minutes. This represents a 50% higher hydrostatic pressure than that supported by the deepest marine mammal, i.e., the Cuvier’s beaked whale [34], for which a maximum diving depth of 3000 m was reported. The same study also revealed that in addition to tolerating high compression, lice supported rapid changes in hydrostatic pressure, which can be thought of as the natural equivalents of the rapid dives and climbs to the surface performed by their hosts [23]. Another significant finding was that seal lice can tolerate hydrostatic pressure by themselves, i.e., they do not need to be associated with the host mammal to do so. It can, therefore, be assumed that this ability is an intrinsic feature of echinophthiriids [23] and that hiding in the hosts’ fur is not crucial to their survival. When penguins are in the water, they trap a blanket of air within their feathers and a warm skin temperature; thus, it appears that their lice do not encounter true marine conditions and can continue to spawn whether the bird is on land or at sea [35].

### 2.6. Ecology

During the 1960s and 1970s, Murray and Kim conducted the first studies on the ecology and life cycles of echinophthiriids. Murray focused on lice from two Antarctic seals, i.e., *A. carlinii* from Weddell seals [31,35] (Murray, 1964; Murray et al., 1965) and *L. macrorhini* from the southern elephant seal [33,35,36,37]; while Kim studied *A. callorhini* and *P. fluctus* from the northern fur seal [22,25,38]. In these pioneering studies, the authors first showed that the reproduction and transmission of echinophthiriids can only occur when their hosts are on land; consequently, their life cycle adjusts precisely to the reproduction cycle of their hosts. The main consequence of this adjustment is a temporal restriction of reproduction, which limits the number of lice generations [27].

As is the case in all lice species, spreading requires close contact between potential hosts. In the particular case of echinophthiriids, transmission occurs during the time that seals spend ashore mating, nursing, molting, or resting [22,28,39]. For most species, it has been reported that the main method of transmission for seal lice occurs from the mother to the newborn pup during nursing [27,38]. However, for other seals, the pattern seems to be different. *Antarctophthirus lobodontis,* from the crabeater seal, is more abundant in juveniles, and lice move between individuals rather than just between mothers and their offspring [32]. As this occurs with reproduction, the strategies of each echinophthiriid species adjust precisely to the particularities of its host species, which reflects a long coevolutionary process.

## 3. Conclusions

The particular biology of seal lice makes them a fascinating example of adaptation. Their long evolutionary history in association with their amphibious hosts has exposed them to selective pressures that no other insect undergoes. The research into the specific morphological, physiological, and behavioral adaptations that enable them to tolerate the harsh environments they encounter during their ectoparasitic life is only just getting underway. A major piece of information that was recently acquired is particularly revealing: the fact that they do not die during the long excursions into the open sea of their deep-diving hosts.

This premise is not as simplistic as it appears. It puts aside the conservative idea that only those remaining on the mainland would somehow survive and wait during most of the year for the return of their hosts ashore for the next reproductive season. With their capacity to survive in extreme environments being confirmed beyond any doubt, we can now focus on the next scientific challenge, i.e., explaining how this is possible. The previous sections presented some hypotheses to be tested and research leads to follow, which should help to decipher the puzzle.

This review helps us identify some key questions to be investigated next, in order to understand better the morphological and physiological adaptations of seal lice to the amphibious life of their host; for example: (1) can seal lice breathe underwater through cuticular diffusion or a plastron? (2) Does the tracheal system completely collapse during dives? (3) Do they reduce their metabolism when submerged, sparing oxygen and energy? (4) Are they capable of keeping an oxygen reserve associated with respiratory pigments? (5) Does high hydrostatic pressure trigger molecular mechanisms that aid in the tolerance of high pressures, as in the synthesis of piezolytes?

Beyond their fascinating biology, seal lice encourage us to forsake the notion that “insects are not made to survive in the ocean”, based on arguments concerning their respiratory system, osmoregulation, or their lack of transparency [40,41]. So far, seal lice have not revealed any unusual structural or physiological adaptations associated with their extraordinary endurance. Their secret appears to be a well-balanced set of traits that they share with a variety of other insects.

So, if insects are able to live in the oceans, a legitimate question is: why are they virtually absent? The study of seal lice suggests that the answer to this question could well not be related to morphological or physiological constraints but probably for evolutionary and/or ecological reasons [41]. Despite the constraints imposed by their biology, we expect that in the near future, these insects will continue to offer more information about their adaptations to marine life.

## Figures and Tables

**Table 1:** Seal-louse associations of the family Echinophthiriidae (Anoplura).

Louse Genus	Species	Host
Antarctophthirus	*A. callorhini*	Northern fur seal
	*A. carlinii*	Weddell seal
	*A. lobodontis*	Crabeater seal
	*A. mawsoni*	Ross seal
	*A. microchir*	Steller, Californian, South American, Australian, and New Zealand sea lion
	*A. ogmorhini*	Leopard seal
	*A. trichechi*	Walrus
Latagophthirus	*La. rauschi*	North American river otter
Lepidophthirus	*Le. macrorhini*	Elephant seals
	*Le. piriformis*	Monk seals
Echinophthirius	*E. horridus*	Northern true seals
Proechinophthirus	*P. fluctus*	Northern fur seal
	*P. zumpti*	Southern fur seals
